# Structural and Functional Characterization of Gray Matter Alterations in Female Patients With Neuropsychiatric Systemic Lupus

**DOI:** 10.3389/fnins.2022.839194

**Published:** 2022-05-02

**Authors:** Li Su, Zhizheng Zhuo, Yunyun Duan, Jing Huang, Xiaolu Qiu, Mengtao Li, Yaou Liu, Xiaofeng Zeng

**Affiliations:** ^1^Department of Rheumatology and Clinical Immunology, Xuanwu Hospital, Capital Medical University, Beijing, China; ^2^Department of Rheumatology and Clinical Immunology, Peking Union Medical College Hospital, Chinese Academy of Medical Sciences & Peking Union Medical College, Beijing, China; ^3^Key Laboratory of Rheumatology and Clinical Rheumatology, National Clinical Research Center for Dermatologic and Immunologic Diseases, Ministry of Education, Beijing, China; ^4^Department of Radiology, Beijing Tiantan Hospital, Capital Medical University, Beijing, China; ^5^Department of Radiology, Xuanwu Hospital, Capital Medical University, Beijing, China

**Keywords:** neurosychiatric systemic lupus, gray matter, resting sate fMRI, female, cerebellar seed-based functional connectivity

## Abstract

**Objective:**

To investigate morphological and functional alterations within gray matter (GM) in female patients with neuropsychiatric systemic lupus (NPSLE) and to explore their clinical significance.

**Methods:**

54 female patients with SLE (30 NPSLE and 24 non-NPSLE) and 32 matched healthy controls were recruited. All subjects received a quantitative MRI scan (FLAIR, 3DT1, resting-state functional MRI). GM volume (GMV), fractional amplitude of low-frequency fluctuation (fALFF), regional homogeneity (ReHo), and degree of centrality (DC) were obtained. Between-group comparison, clinical correlation, and discrimination of NPSLE from non-NPSLE were achieved by voxel-based analysis, cerebellar seed-based functional connectivity analysis, regression analysis, and support vector machine (SVM), respectively.

**Results:**

Patients with NPSLE showed overt subcortical GM atrophy without significantly abnormal brain functions in the same region compared with controls. The dysfunction within the left superior temporal gyri (L-STG) was found precede the GM volumetric loss. The function of the nodes in default mode network (DMN) and salience network (SN) were weakened in NPSLE patients compared to controls. The function of the cerebellar posterior lobes was significantly activated in non-NPSLE patients but attenuated along with GM atrophy and presented higher connectivity with L-STG and DMN in NPSLE patients, while the variation of the functional activities in the sensorimotor network (SMN) was the opposite. These structural and functional alterations were mainly correlated with disease burden and anti-phospholipid antibodies (aPLs) (r ranges from -1.53 to 1.29). The ReHos in the bilateral cerebellar posterior lobes showed high discriminative power in identifying patients with NPSLE with accuracy of 87%.

**Conclusion:**

Patients with NPSLE exhibit both structural and functional alterations in the GM of the brain, which especially involved the deep GM, the cognitive, and sensorimotor regions, reflecting a reorganization to compensate for the disease damage to the brain which was attenuated along with pathologic burden and cerebral vascular risk factors. The GM within the left temporal lobe may be one of the direct targets of lupus-related inflammatory attack. The function of the cerebellar posterior lobes might play an essential role in compensating for cortical functional disturbances and may contribute to identifying patients with suspected NPSLE in clinical practice.

## Introduction

Systemic lupus erythematosus (SLE) is a chronic autoimmune disorder involving multiple organ systems, typically presents in females of childbearing age, with the incidence ratio of female to male is around 9:1 (Mok et al., 1999). The role of female reproductive hormones in the development of the disease has been reported (Costenbader et al., 2007). Neuropsychiatric systemic lupus (NPSLE) is one of the most common manifestations of lupus, affecting 21–95% of patients with SLE and related to high disability and mortality (Pamfil et al., 2015). The central nervous system is involved in approximately 90% of patients with NPSLE, with various clinical manifestations, including cognitive impairment, headache, mood disorders, cerebrovascular disease, psychosis, seizures, and acute confusional state, etc. (Pamfil et al., 2015).

The pathogenesis of NPSLE is not yet fully clear, but several mechanisms are implicated, including autoimmune inflammatory neuronal damage, vasculitis and vasculopathy with ischemia, precocious atherosclerosis, and embolisms (Cohen et al., 2017; Gelb et al., 2018). The diagnosis of NPSLE in clinical practice still requires the judgment of experienced physicians. Correct attribution of neuropsychiatric events to NPSLE or an alternative etiology is still a challenge, considering the absence of a diagnostic gold standard. Magnetic resonance imaging (MRI) of the brain has been applied to SLE for years, but the findings are nonspecific; the most common presentations of conventional MRI are cerebral atrophy (15–20%), diffused white matter (WM) lesions or hyperintensities (30–75%), focal lesions after stroke, etc (Sarbu et al., 2015). However, more than 40% of patients with NPSLE showed no remarkable changes on conventional MRI (Luyendijk et al., 2011). Hence, advanced brain imaging techniques (e.g., structural and resting state functional MRI) have been applied into this field to successfully characterize the brain microstructural and functional abnormalities, to study *in vivo* neural mechanisms of neurologic and psychiatric manifestations of the disease invisible with only structural imaging, attempting to help classify and evaluate patients with suspected NPSLE in clinical practice (Sarbu et al., 2017). Resting state (RS) functional connectivity (FC) abnormalities have been reported both in NPSLE patients and non-NPSLE patients (Nystedt et al., 2019; Bonacchi et al., 2020; Cao et al., 2021), suggesting reorganizations of the neuronal networks may take place even before the onset of neuropsychiatric symptoms, and may be adaptive or maladaptive to the brain functional impairments.

Meanwhile, previous neuroimaging studies suggest that lupus patients have characteristic subcortical and regional gray matter atrophy when compared to controls (Jung et al., 2010; Kalinowska-Łyszczarz et al., 2018), and that these group differences may be more significant in NPSLE patients. However, other functional MRI research of SLE patients indicated an apparent lack of overlap between gray matter volume reduction and functional alterations. The different patterns of relationship between the structure and function of brain found in the disease worth exploring more deeply (Lin et al., 2011).

In this context, by using structural and resting state functional MRI, we aimed to investigate the morphological and functional alterations of the gray matter and their possible inter-relationship in female patients with NPSLE, and to explore their potential clinical significance. This approach increased the homogeneousness of the enrolled subjects and the accuracy of the results.

## Materials and Methods

### Participants

Seventy-four female patients (including 40 NPSLE and 34 non-NPSLE) were randomly recruited from the Department of Rheumatology and Immunology in Peking Union Medical College Hospital, fulfilling at least four of the American College of Rheumatology (ACR) classification criteria for SLE (Smith and Shmerling, 1999) between Jan 2017 and Dec 2018. Thirty-six female age-matched healthy controls (HCs) were enrolled. Primary CNS NPSLE manifestations were defined according to the ACR definition and the Systemic Lupus International Collaborating Clinics (SLICC) model B criteria (Bortoluzzi et al., 2018). All the clinical information of patients with SLE was verified by an experienced rheumatologist and an experienced neurologist. The diagnosis of cognitive disorder and mood disorder were made by the neurologist according to the routine screening tests [Mini-Mental State Exam (MMSE), Montreal Cognitive Assessment (MoCA), Hamilton Anxiety Scale (HAMA), and Hamilton Depression Scale (HAMD)]. The inclusion criteria also included age between 18 and 65 years and right handedness. The exclusion criteria were as follows: (Mok et al., 1999) taking psychoactive medication or alcohol/drug abuse; (Costenbader et al., 2007) any current or past diagnosed primary mental illness; (Pamfil et al., 2015) secondary NPSLE due to infections, electrolyte disturbances, hypertension, or other causes; (Cohen et al., 2017) any evident MRI lesions in the HCs; (Gelb et al., 2018) further contraindications to MRI scan; and (Sarbu et al., 2015) poor MRI image quality, e.g., overt motion and susceptibility artifacts, and low signal-to-noise ratio. From the original cohort, 20 patients with SLE and 4 HCs were excluded due to Mok et al. (1999) incomplete records of medical history (*n* = 6); (Costenbader et al., 2007) secondary NPSLE (*n* = 5); (Pamfil et al., 2015) remittent mild headache as the single neuropsychiatric symptom (*n* = 3); and (Cohen et al., 2017) poor MRI data quality (*n* = 10). The remaining 30 patients with primary CNS NPSLE with at least one classified neuropsychiatric symptom, 24 patients with non-NPSLE and 32 healthy volunteers were finally enrolled. All the patients had received treatment with steroids and immunosuppressors. Disease activity was assessed using the Systemic Lupus Erythematosus Diseases Activity Index 2000 (SLEDAI-2k) scores. Accumulative disease damage was assessed with the Systemic Lupus International Collaborating Clinics/American College of Rheumatology (SLICC/ACR) damage index (SDI) scores. The demographics, clinical data (disease duration, manifestations of SLE, current medications, and immunological data) were registered. The interval between clinical evaluations and MRI scans was within 7 days. The study protocol was approved by the Ethics Committee at the Peking Union Medical College Hospital. All participants gave their written informed consent.

### Image Acquisition

The conventional MR sequences [T2 and fluid-attenuated inversion recovery (FLAIR)], high-resolution T1-weighted imaging [3DT1], and resting state functional MRI [rs-fMRI] were performed on a 3.0-Tesla MR system (Siemens Magnetom Trio Tim System, Siemens Healthcare GmbH, Erlangen, Germany) using a 32-channel head coil. Axial T2-weighted images and FLAIR images with 4-mm slice thickness were acquired for lesion identification. High-resolution anatomical images were acquired using T1-weighted three-dimensional volumetric magnetization-prepared rapidly acquired gradient-echo (MPRAGE) sequence: repetition time (TR) = 1600 ms; echo time (TE) = 2.13 ms; flip angle (FA) = 9°; inversion time (TI) = 1000 ms; in-plane resolution 1 × 1 mm^2^; slice thickness = 1 mm; matrix = 256 × 224; 176 axial slices. Rs-fMRI data were collected using a gradient rapid echo-echo planar imaging (GRE-EPI) sequence: TR = 2000 ms; TE = 30 ms; FA = 90°; in-plane resolution = 3.5 × 3.5 mm^2^; slice thickness = 3 mm; slice gap = 1 mm; matrix = 64 × 64; 35 axial slices. The MRI scans of the patients with SLE were acquired at least 4 weeks from the last relapse and treatment to minimize their confounding effects on the following analysis.

### Magnetic Resonance Imaging Image Processing

The fMRI images were preprocessed by using DAPARSF (Data Processing Assistant for Resting-State fMRI, Advanced Edition^1^). Preprocessing steps include removing the first 10 time points, slice timing correction, realigning fMRI volumes, reorienting fMRI and T1 images, coregistering the structural T1 image to functional MRI image, segmenting the structural T1 with DARTEL (Diffeomorphic Anatomical Registration Through Exponentiated Lie Algebra) and then warping these images into Montreal Neurological Institute (MNI) space, regressing the nuisance covariates (including signal linear drift, head motion parameters, mean signals within white matter and CSF), warping the processed fMRI images into MNI space with the normalization parameters derived from the structural T1 segmentation and normalization, and resampling the fMRI voxel into 3 mm × 3 mm × 3 mm. In our preprocessing steps, smoothing was not carried out to preserve the signal details.

The fractional low frequency amplitude (fALFF) within the 0.01-0.1 Hz band was calculated, and then the fMRI signals were filtered with the frequency band of 0.01-0.1 Hz to reflect the low-frequency oscillator fluctuations of resting state fMRI signals. The regional homogeneity (ReHo) to measure the similarity of time series within local brain areas and degree centrality (DC) to measure the importance of local brain areas in the functional connectivity were obtained by using the filtered images. Z-score maps of all the parameter images were obtained and smoothed by a 4-mm full width at half maximum Gaussian kernel for the following voxel-based statistical analysis. Additionally, the segmented and normalized gray matter (GM) images in MNI space were modulated and smoothed for voxel-based morphometry (VBM) analysis.

The CONN (Whitfield-Gabrieli and Nieto-Castanon, 2012) v.20.b toolbox^2^ is used for resting-state functional connectivity analysis. Pre-processing of the data used the default pipeline of CONN included discard the first 10 time points, slice-timing correction, functional realignment and unwarping, structural segmentation, functional and structural normalization in the MNI-space (normalization of the co-registered T1 image and EPI volumes with a voxel size of 2 × 2 × 2mm), functional outlier detection (ART-based scrubbing) and smoothing (8-mm FWHM Gaussian filter). Then, the toolbox step to a denoising procedure: the confounding effects such as the white matter, cerebrospinal fluid, realignment results, scrubbing results, and the rest were regressed out of the fMRI time series, and after that, the data were bandpass-filtered with the default CONN values (0.008–0.09 Hz) and linear detrended.

### Seed Based Connectivity Analysis (Seed-To-Voxel Analysis)

According to FSL Harvard-Oxford atlas in CONN, bilateral cerebellar Crus I & Crus II and only right cerebellar Crus II were used as the seeds, respectively. Their BOLD response was correlated with those of each voxel in the rest of the brain.

### Statistical Analysis

The statistical analysis was performed using SPSS (SPSS for Windows, version 25.0; IBM, Armonk, NY, United States), the statistics toolbox in MATLAB (MATLAB 2019a) and Statistical Parametric Mapping (SPM12^3^).

The values are expressed as the mean and standard deviation (SD) for normally distributed variables and median and interquartile range (IQR) for parameters without a normal distribution.

One-way ANOVA and *post hoc* comparison and Student’s *t*-test were used for variables with a normal distribution. The Wilcoxon and Kruskal-Wallis test, *post hoc* analysis and Mann-Whitney U test were used for variables that were not normally distributed. Multiple comparisons were performed by Bonferroni correction. P value < .05 was deemed statistically significant.

For voxel-based statistical analysis of GM structural and functional measures, nonparametric one-way ANOVA [permutation test with 5000 permutations and familywise error (FWE) correction for multiple comparisons with p < 0.05] with age and total intracranial volume [TIV, only for GM volume (GMV)] as covariates were first performed, followed by nonparametric two-sample analysis (permutation test with 5000 permutations and FWE correction for multiple comparisons p < 0.05) to compare each pair of groups.

For seed-to-voxel analysis, one-way ANOVA was used to compare the differences in functional connectivity between NPSLE, non-NPSLE and HCs groups. Then, multiple comparisons were adjusted by applying the correction of False discovery rate (FDR) (p < 0.05).

Linear regression analyses were performed to find the associations between the MRI and clinical features with adjustment for age for SLE patients (including both NPSLE and non-NPSLE).

Logistic regression analysis was performed to evaluate the ability [by sensitivity, specificity, and area under the curve (AUC)] of structural and functional MRI measures to distinguish between patients with NPSLE and patients with non-NPSLE.

### Support Vector Machine for Discriminating Patients With Neuropsychiatric Systemic Lupus From Patients With Non-neuropsychiatric Systemic Lupus

Support vector machine with linear kernel (SVM, using libsvm^4^) was adopted to identify patients and further distinguish different types of patients with SLE by using structural and functional features, which showed statistically significant differences between groups. Multivariate logistic regression was first used for feature selection. Leave-one-out cross-validation was adopted to train and evaluate the SVM model. Accuracy, sensitivity, specificity, precision, recall, and F1-score were used to evaluate the performance of the classification.

## Results

### Demographic Characteristics and Clinical Findings

All demographic and clinical characteristics are summarized in Table 1. There was no significant difference in disease duration, SLEDAI scores, the rate of patients with SLEDAI ≥ 5, anti-ribosomal P protein antibody, antiphospholipid antibodies (aPLs), traditional vascular risk factors or current medication between patients with NPSLE and non-NPSLE (all *p* > 0.05). However, patients with NPSLE showed higher Systemic Lupus International Collaborating Clinics/American College of Rheumatology(SLICC/ACR) Damage Index (SDI) scores compared to the patients with non-NPSLE, which was mainly due to the neurological involvement (*p* < 0.05).

**TABLE 1:** Demographic and clinical characteristics of SLE patients with and without neuropsychiatric manifestations.

	non-NPSLE	NPSLE	P value
	(*n* = 24)	(*n* = 30)	
Age, mean (SD), years	29.1 (10.0)	32.5 (12.8)	0.30
Duration, median (IQR), years	28.0 (10.5, 54.0)	66.0 (12.0, 168.0)	**0.060**
SLEDAI score, median (IQR)	4.0 (1.0, 7.5)	6.0 (3.0, 12.0)	**0.061**
SLEDAI score ≥ 5, n (%)	9 (38%)	18 (62%)	0.10
Non-neurological SLEDAI score, median (IQR)	4.0 (1.0, 7.5)	4.0 (2.0, 8.0)	0.55
SLICC SDI score, median (IQR)	0.0 (0.0, 0.0)	1.0 (0.0, 1.0)	** < 0.001**
Active NP, n (%)		10 (33.3%)	
Period between 1st NP event to scanning, median (IQR), months		32.5 (40.4)	
Period between last NP event to scanning, median (IQR), months		9.2 (16.4)	
**Cumulative organ system involvement, n (%)**			
Cutaneous	15 (63%)	22 (73%)	0.56
Vasculitis	6 (25%)	6 (20%)	0.75
Articular	12 (50%)	14 (47%)	1.00
Serositis	6 (25%)	10 (33%)	0.56
Renal	12 (50%)	18 (60%)	0.58
Hematologic	18 (75%)	19 (63%)	0.39
Interstitial pneumonia	2 (8%)	6 (20%)	0.28
Cardiac	0 (0%)	3 (10%)	0.25
**Manifestations of NP, n (%)**			
Seizure disorder		13(43.3%)	
Cerebrovascular events		5(16.7%)	
Acute confusional state		5(16.7%)	
Psychosis		6(20.0%)	
Cognitive disorder		12(40.0%)	
Mood disorder		9(30.0%)	
Severe headache		7(23.3%)	
Demyelinating syndrome		9(30.0%)	
**Laboratory findings, n (%)**			
Anti-dsDNA (+)	12 (50%)	18 (60%)	0.58
Anti-ribosomal P (+)	8 (33%)	15 (50%)	0.27
aPLs (+)	5 (21%)	12 (40%)	0.15
Hypocomplementemia	19 (79%)	28 (93%)	0.22
**Complications, n (%)**			
Sjogren’ syndrome	2 (8%)	7 (23%)	0.27
Hashimoto’s disease	1 (4%)	5 (17%)	0.21
Smoking	0 (0%)	0 (0%)	
Hypertension	1 (4%)	7 (23%)	0.063
Diabetes	1 (4%)	1 (3%)	1.00
Dyslipidemia	0 (0%)	4 (13%)	0.12
**Current medication, n(%)**			
Glucocorticoids	21 (88%)	29 (97%)	0.31
Cumulative dose of steroids, median (IQR)	11.2 (3.9, 14.6)	11.4 (7.2, 29.4)	0.16
Low dose of steroids (Pred < 10mg/d)	15 (63%)	15 (52%)	0.58
*DMARDs*	20 (83%)	24 (83%)	1.00
Anticoagulants/antiplatelets	4 (17%)	9 (31%)	0.34
Lipid lowering agents	2 (8%)	9 (31%)	0.086
Vasodilators	0 (0%)	4 (14%)	0.12
**cMRI Imaging**			
Lesion Volume, median (IQR)	0.0 (0.0, 1164.0)	2188.0 (0.0, 6176.0)	**0.002**

*Values are mean ± SD, median (IQR), or number (%).*

*P values are for Wilcoxon’s rank sum test on continuous variables and for Fisher’s exact test on categorical variables.*

*Include Cyclophosphamide, Mycophenolate Mofetil, Azathioprine, Methotrexate, Ciclosporin, and Tacrolimus.

*SD, standard deviation; IQR, interquartile range; SLE, systemic lupus erythematosus; anti-dsDNA, anti-double strand DNA antibody; aPLs, anti-phospholipid antibodies; SLEDAI, Systemic Lupus Erythematosus Disease Activity Index; SLICC SDI, Systemic Lupus International Collaborating Clinics/American College of Rheumatology Damage Index; DMARDs, disease modifying antirheumatic drugs; cMRI, conventional MRI.*

*The bold values mean p < 0.05 or nearly to 0.05.*

The current study included 10(33.3%) patients with active NPSLE. The median time interval between the first NP event to the imaging assessment was 32.5 (40.4) months, while the median time interval between the last NP event to the imaging assessment was 9.2 (16.4) months. The neuropsychiatric manifestations of patients with NPSLE included seizure disorders (*n* = 13, 43.3%), cognitive disorder (*n* = 12, 40%), demyelinating syndrome (*n* = 9, 30%), mood disorder (*n* = 9, 30%), severe headache (*n* = 7, 23.3%), psychosis (*n* = 6, 20.0%), acute confusional state and cerebrovascular events (*n* = 5, 16.7%, respectively).

On conventional MRI, patients with NPSLE showed significantly increased lesion volume, when compared with patients with non-NPSLE [0.0 (0.0, 1164.0) vs 2188.0 (0.0, 6176.0), *p* = 0.002].

### Gray Matter Volumetric Alterations in Systemic Lupus Erythematosus

**FIGURE 1:**
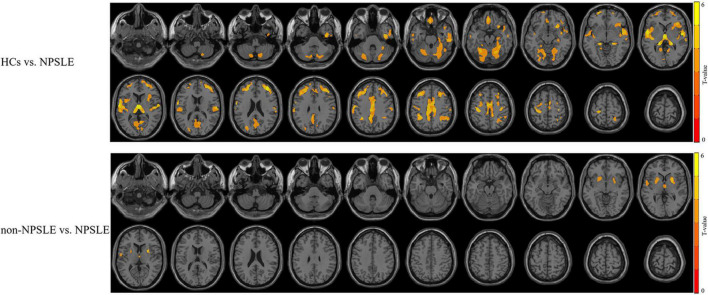
The different patterns of GM atrophy in patients with NPSLE and non-NPSLE. The colored bar indicates the statistical T distribution between groups.

Compared to HCs, patients with non-NPSLE presented no significant GM atrophy, while patients with NPSLE presented widespread GM atrophy in the cortical cortex including the frontal (e.g., rectus gyrus and precentral gyrus), temporal (e.g., superior/inferior temporal gyrus), parietal (e.g., postcentral and precuneus gyrus) and occipital (e.g., fusiform, lingual gyrus and calcarine) cortex, subcortical nuclei (e.g., thalamus, hippocampus, and putamen) and cerebellum (Figure 1 and Table 2).

**TABLE 2:** Statistically significant structural and functional alterations in SLE patients using voxel-based analysis.

MR features	Between groups	Brain areas	cluster size (voxels)	Peak MNI coordinate			Peak T
				X	Y	Z	
Gray matter volume	HCs vs NPSLE	Right cerebellar anterior and posterior lobe/bilateral thalamus	8392	–1.5	–10.5	7.5	5.56
		Left cerebellar anterior and posterior lobe	4443	–28.5	–55.5	–18	4.55
		Right inferior and middle temporal gyrus/right fusiform gyrus	1972	37.5	–10.5	–36	5.03
		Left inferior temporal gyrus	285	–57	–12	–18	3.95
		Right insula/right superior temporal gyrus	6169	27	22	–13	5.13
		Left rectus gyrus	1123	–3	37.5	–22.5	4.47
		Left superior and middle frontal gyrus	3990	–31	29	46	4.54
		Left superior temporal gyrus/left postcentral gyrus/left insula	6156	–53	–1	1	5.17
		Right superior and middle frontal gyrus	4267	36	40	26	5.17
		Bilateral cingulum and bilateral precuneus	10240	–2	–6	49	5.09
		Left middle occipital gyrus	424	–24	–75	22.5	4.1696
		Right middle cingulum	225	–1.5	39	31.5	3.952
		Left inferior parietal gyrus	608	–35	–46	41	4.53
		Right superior and inferior parietal gyrus	1314	28.5	–55.5	45	4.6806
		Right superior and middle frontal gyrus	268	25.5	–3	54	3.9186
	Non-NPSLE vs NPSLE	Left putamen	739	–22.5	7.5	1.5	4.0225
		Right putamen	701	24	6	6	4.4821
		Bilateral thalamus	269	3	–10.5	3	3.7348
		Left superior temporal gyrus	390	–55.6	–6	6	4.0848
ReHo	HCs vs non-NPSLE	Left cerebellar posteiro lobe	48	–48	–54	–45	–3.7567
		Left superior temporal; pole	73	–39	9	–27	5.0354
		Right precentral gyrus	36	9	–21	78	3.2704
		Left postcentral gyrus	50	0	–30	72	3.4827
	HCs vs NPSLE	Left superior temporal pole	40	–39	15	–21	3.9901
		Right Fusiform/right lingual gyrus	51	27	–69	6	–3.9288
		Right insula	42	42	15	–6	4.1044
		Left anterior Cingulum	41	–3	42	–3	3.7632
		Vermis_4_5	32	–3	–35	–1	3.6127
	Non-NPSLE vs NPSLE	Left cerebellar posterior lobe	150	–51	–54	–45	4.1608
		Right cerebellar posterior lobe	59	33	–51	–42	3.6234
DC	HCs vs non-NPSLE	Right postcentral gyrus	30	30	–36	72	3.3265
	HCs vs NPSLE	Right cerebellar posterior lobe	30	6	–93	–36	–3.4111
fALFF	HCs vs non-NPSLE	Left inferior occipital gyrus	32	–30	–81	–12	–3.5115
		Left postcentral gyrus	120	–3	–33	75	3.5695
	HCs vs NPSLE	Left superior medial frontal gyrus/left anterior cingulum	108	–3	48	33	4.4221
		Bilateral middle cingulum_	201	1	23	32	4.66
	non-NPSLE vs NPSLE	left Postcentral Gyrus	39	–15	–45	72	–3.1991

*Peak T, peak T value.*

Compared to patients with non-NPSLE, patients with NPSLE presented GM atrophy in the left superior temporal gyrus, right thalamus, and bilateral putamen (Figure 1 and Table 2).

### Gray Matter Functional Alterations in Systemic Lupus Erythematosus

**FIGURE 2:**
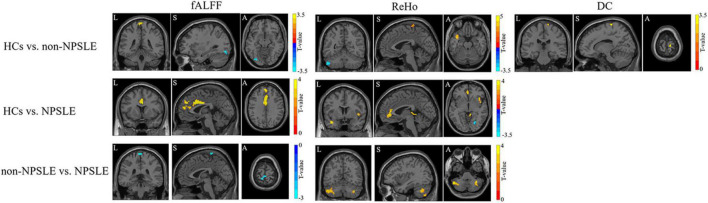
The fALFF, ReHo and DC changes in patients with NPSLE and non-NPSLE. The colored bar indicates the statistical T distribution between groups.

Compared to HCs, patients with non-NPSLE presented increased fALFF in the left inferior occipital lobe and decreased fALFF in the bilateral postcentral and paracentral lobules; patients with NPSLE presented decreased fALFF in the left medial superior frontal gyrus, left anterior cingulum, and bilateral middle cingulate. Compared to non-NPSLE, patients with NPSLE presented increased fALFF in the bilateral postcentral gyrus (Figure 2 and Table 2).

Compared to HCs, patients with non-NPSLE presented increased ReHo in the left cerebellar posterior lobe (L-Crus II, L-Crus I) and decreased ReHo in the left superior temporal gyrus, left postcentral gyrus, and right precentral gyrus; patients with NPSLE presented increased ReHo in the right fusiform and lingual gyrus and decreased ReHo in the left anterior cingulum gyrus, left superior temporal gyrus, right insula, and cerebellar vermis. Compared to non-NPSLE, patients with NPSLE presented decreased ReHo in the bilateral cerebellar posterior lobes (Crus II, Crus I) (Figure 2 and Table 2).

Compared to HCs, patients with non-NPSLE presented decreased DC in the right postcentral gyrus. No difference was found between HCs and patients with NPSLE or between patients with non-NPSLE and those with NPSLE (Figure 2 and Table 2).

### Cerebellar Seed-Based Functional Connectivity Alterations in Systemic Lupus Erythematosus

According to the results from the structural and functional imaging data analysis mentioned above, we found that the cerebellar posterior lobes might play a crucial role in the compensation for the disease damage. We next did the seed-based functional connectivity analysis with using the cerebellar posterior lobes (Crus I, Crus II) as the seeds, to further investigate the potential interaction of the cerebellar posterior lobes with other cognitive networks in SLE.

**FIGURE 3:**
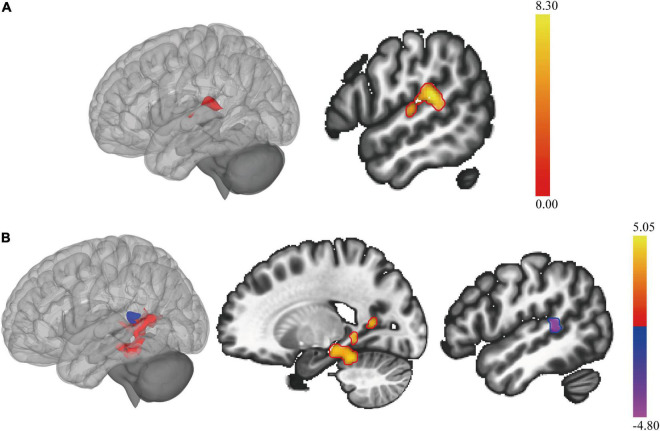
The cerebellar seed-based resting state functional connectivity (RS FC) changes in patients with NPSLE and non-NPSLE. **(A)**: Clusters of abnormal RS FC between subgroups of patients with SLE, as the bilateral cerebellar posterior lobes (cerebellar Crus I & Crus II) being the seed. **(B)**: Clusters of abnormal RS FC between subgroups of patients with SLE, as the right cerebellar Crus II being the seed. The colored bar indicates the statistical T distribution between groups.

Figure 3 and Table 3 summarized the voxel-wise differences of RS FC between subgroups, with the cerebellar posterior lobes as the seeds. Compared to non-NPSLE patients, NPSLE patients exhibited hyperconnectivity between the bilateral Crus I & II region and the left-posterior superior temporal gyrus (L-pSTG), left planum temporale and left parietal operculum. Specifically, with a seed placed at the right cerebellar Crus II, an essential node for the cerebellum network, the NPSLE group had increased FC of the posterior cingulate gyrus, precuneous cortex, left posterior temporal fusiform cortex and left posterior parahippocampal gyrus within the posterior DMN, but reduced FC of the L-pSTG and left planum temporale as compared to the non-NPSLE group.

**TABLE 3:** Clusters of abnormal RS FC between HC and SLE patients and between subgroups of SLE patients, as the parts of the cerebellar posterior lobes (Crus I, Crus II) being the seeds.

Contrast	Clusters	Size	X	Y	Z	F	P_*FDR*_
**Seed: bilateral cerebellar Crus I & Crus II**							
NPSLE>HCs							NS
non-NPSLE>HCs							NS
NPSLE>non-NPSLE	left planum temporale left parietal operculum cortex L- pSTG	274	–54	–32	+16	7.35	0.018
**Seed: right cerebellar Crus II**							
NPSLE>HCs							NS
non-NPSLE>HCs							NS
NPSLE>non-NPSLE	Posterior cingulate gyrus L cerebellum 4 5 Precuneous cortex	339	–12	–42	2	4.97	0.012
	Posterior cingulate gyrus	259	20	–44	0	4.60	0.022
	L cerebellum 4 5 Posterior temporal fusiform cortex L posterior Parahippocampal gyrus	213	–60	–40	–10	–5.39	0.025
NPSLE<non-NPSLE	L planum temporale L-pSTG	233	–22	–40	10	4.93	0.023

*L, left; L-pSTG, left posterior superior temporal gyrus.*

### Correlations of Magnetic Resonance Imaging Indices With Clinical Variables

As shown in Table 4, lesion volume presented significant negative correlations with GM volume (GMV) in the bilateral putamen and GMV in the right thalamus and ReHo in the left cerebellum.

**TABLE 4:** The clinical associations of MRI measurements with clinical variables using linear regression in SLE patients (including both NPSLE and non-NPSLE).

Features	Brain regions	Disease duration (months)	SLEDAI scores	SDI scores	Cumulative steroid dose (g)	Lesion volume	Hypertension	aPLs
GMV	right cerebellum/fusiform/lingual/ hippocampus/bilateral thalamus			–0.37 (0.035)				
	Left cerebellum/fusiform/lingual gyrus			–0.46 (0.008)				
	right inferior frontal/precentral/postcentral/ superior temporal/insula/putamen			–0.40 (0.021)				
	left inferior frontal/precentral/postcentral/ superior temporal/insula			–0.33 (0.049)				
	left putamen					–0.49 (< 0.001)		
	right putamen					–0.40 (0.001)		
	right thalamus			–0.44 (0.008)		–0.32 (0.028)		
	left superior temporal			–0.36 (0.020)				
fALFF	whole brain							–0.14 (0.037)
	left inferior occipital		–0.49 (0.003)					–0.12 (0.024)
ReHo	whole brain							–0.16 (0.039)
	left cerebellum	–1.53 (0.001)			1.29 (0.003)			
	right precentral	1.15 (0.032)			–1.17 (0.020)			
	left postcentral/paracentral lobule						0.32 (0.033)	
	left anterior cingulum							0.14 (0.024)
	left cerebellum	–1.31 (0.005)			0.87 (0.041)	–0.41 (0.033)		
DC	right postcentral	0.75 (0.034)	0.36 (0.03)		–0.87 (0.009)		0.31 (0.002)	
	cerebellar posterior lobe			0.55 (0.009)				

*The results are presented with the regression coefficients and the corresponding p values. Statistical significance of two-sided p < 0.05 was adopted.*

Disease duration presented negative correlations with ReHo in the left cerebellum and positive correlations with ReHo in the right precentral cortex and DC in the right postcentral gyrus.

Systemic Lupus Erythematosus Diseases Activity Index (SLEDAI) scores presented a positive correlation with DC in the right postcentral cortex and a negative correlation with fALFF in the left inferior occipital gyrus.

SDI scores presented negative correlations with GMV in the right cerebellum/fusiform/lingual/hippocampus/bilateral thalamus, GMV in the left cerebellum/fusiform/lingual gyrus, GMV in the right inferior frontal/precentral/postcentral/superior temporal/insula/putamen, GMV in the left inferior frontal/precentral/postcentral/superior temporal/insula, GMV in the right thalamus, and GMV in the left superior temporal gyrus and a positive correlation with DC in the cerebellar posterior lobe.

Cumulative doses of steroids presented significant positive correlations with ReHo in the left and right cerebellum and negative correlations with ReHo in the right precentral gyrus and DC in the right postcentral gyrus.

Hypertension was positively correlated with ReHo in the left postcentral/paracentral lobule and DC in the right postcentral gyrus.

Anti-phospholipid antibodies (aPLs) presented a mildly negative correlation with fALFF and ReHo at the whole brain level, fALFF in the left inferior occipital region and a mildly positive correlation with ReHo in the left anterior cingulum.

### Logistic Analysis for the Discrimination of Patients With Neuropsychiatric Systemic Lupus From Non-neuropsychiatric Systemic Lupus

**FIGURE 4:**
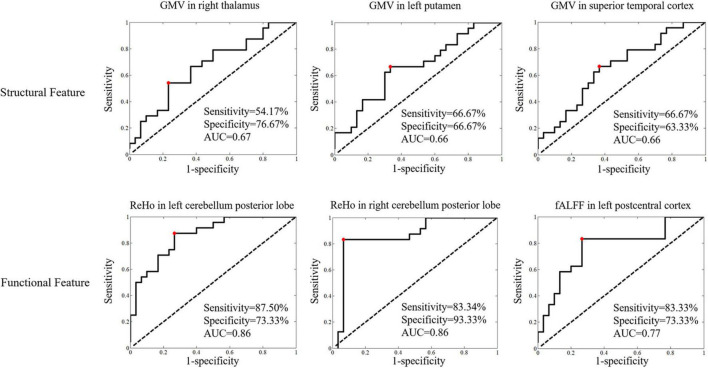
The logistic regression analyses of the structural and functional features in statistically significant brain regions for discrimination of patients with NPSLE from those with non-NPSLE (features with the top 3 leading AUCs are presented). Classificational sensitivity, specificity and AUC are presented at the cut-off point (red).

For the discrimination of patients with NPSLE from non-NPSLE, logistic analysis results showed structural features, with the top 3 leading AUCs being GMV in the right thalamus, left putamen and left superior temporal, and functional features with the top 3 leading AUCs being ReHo in the bilateral cerebellum and left postcentral gyrus (Figure 4).

### Support Vector Machine Classification for the Discrimination of Patients With Systemic Lupus Erythematosus From Healthy Controls and Patients With Neuropsychiatric Systemic Lupus From Non-neuropsychiatric Systemic Lupus

As shown in Table 5, the structural and functional features were selected by logistic regression with coefficients of p < 0.005 and p < 0.001 to investigate the robust features. The classification performance had accuracies of 94.44% and 87.04% for features with p < 0.005 and p < 0.001 for the discrimination of patients with NPSLE from patients with non-NPSLE, respectively. The most robust MRI features were ReHo in the bilateral cerebellar posterior lobes.

**TABLE 5:** SVM classification results for the diagnosis of SLE from HCs and NPSLE from non-NPSLE using statistically significant structural and functional features selected by logistic regression with *p* < 0.005 and *p* < 0.001, respectively.

	Feature numbers	Accuracy (%)	Sensitivity (%)	Specificity (%)	Precision (%)	Recall (%)	F1 score
**MRI features (logistic regression with *p* < 0.005)**							
HCs vs non-NPSLE+NPSLE	9	97.67	98.15	96.88	98.15	98.15	0.98
HCs+non-NPSLE vs NPSLE	10	93.02	96.67	91.07	85.29	96.67	0.91
HCs vs non-NPSLE	12	94.64	875	100	100	87.50	0.93
HCs vs NPSLE	9	100	100	100	100	100	100
non-NPSLE vs NPSLE	5	94.44	96.67	91.67	93.55	96.67	0.95
**MRI features (logistic regression with *p* < 0.001)**							
HCs vs non-NPSLE+NPSLE	9	97.67	98.15	96.88	98.15	98.15	0.98
HCs+non-NPSLE vs NPSLE	8	95.35	93.33	96.43	93.33	93.33	0.93
HCs vs non-NPSLE	5	94.64	91.67	96.88	95.65	91.67	0.94
HCs vs NPSLE	8	100	100	100	100	100	100
non-NPSLE vs NPSLE	2	87.04	90.00	83.33	87.10	90.00	0.89

## Discussion

In this study, we adopted a well-validated quantitative MRI approach (3D T1, T2/FLAIR, and rs-fMRI) to investigate the structural and functional characteristics of GM in patients with NPSLE compared with patients with non-NPSLE and HCs. Then, we correlated MRI abnormalities with clinical variables to study the clinical relevance of our findings. Finally, we introduced SVM, the more advanced discriminative approach, to identify potential MRI imaging biomarkers to assist the diagnosis of NPSLE.

In accordance with previous MRI imaging studies, we found widespread GM atrophy in patients with NPSLE (Appenzeller et al., 2007; Jung et al., 2010; Piga et al., 2015; Liu et al., 2018), with significant subcortical GM (the right thalamus and bilateral putamen) atrophy as compared to patients with non-NPSLE, as showed in Figure 1 and Table 2. SLE is bound to cause cerebral atrophy through autoantibody and cytokine induced vascular damage, blood-brain barrier (BBB) impairment, inflammatory neurotoxicity, and other uncovered mechanisms (Sibbitt et al., 2010; Prechl and Czirjak, 2015; Cohen et al., 2017; Schwartz et al., 2019). However, subcortical GM atrophy is not specific for NPSLE: it can also be found in other neuropsychiatric diseases, such as Parkinson’s disease, Alzheimer’s disease, multiple sclerosis, depression, and autism (Orhun et al., 2019). Subcortical GM atrophy in NPSLE might be a mixture of several mechanisms, including vulnerability of this territory to hemodynamic and hypoxic impairment (Chiang et al., 2019; Eslami et al., 2019). It is putatively linked to cognitive decline. However, Kalinowska-Łyszczarz et al. (2018) found no correlations between subcortical atrophy and cognitive deficits in SLE. They concluded the cognitive impairment in SLE is independent of brain atrophy or lesion volume. Notably, in our present study, we also found no significant functional alterations in basal ganglia or thalamus in SLE patients, suggesting that the function of subcortical nuclei is relatively intact in SLE, similar with the results we previously found in neuromyelitis optica (NMO) (Liu et al., 2015). The dissociation of morphological and functional alterations in GM within this region in SLE may further support the hypothesis of brain reorganization to compensate for the functional impairments caused by neuronal injury in SLE.

In what follows, as showed in Figure 2 and Table 2, we found that in patients with non-NPSLE, the function of the left superior temporal gyri (L-STG) within default mode network (DMN) was decreased without detectable volumetric reduction, while in patients with NPSLE, the functional impairment of the same region was conspicuous with significant atrophy. L-STG is involved in limbic system connecting closely with hippocampus. These regions play crucial roles in social cognition and emotion regulation. Microstructural and functional alterations of this area were previously reported in patients with autoimmune encephalitis (e.g., anti-NMDAR) with schizophrenia-like psychiatric manifestations as the initial presentation (Bost et al., 2016). Meanwhile, prior studies using mouse models and *in vitro* experiments demonstrated that anti-double stranded DNA antibody and anti-ribosomal P protein antibody could cross react with neuron surface receptors including NMDARs (DeGiorgio et al., 2001), mainly injuring the hippocampus, inducing neuronal death and leading to cognitive disorders and memory loss. Taken together, our data thus verified *in vivo* the L-STG is one of the potential neural substrates of neuropsychiatric impairment in patients with SLE (Kowal et al., 2006; Schwarting et al., 2019). Besides, choric neurological histopathological lesions characterized by non-specific focal vasculopathy have already been found in the brain tissue of patients with non-NPSLE, while in that of patients with NPSLE, it progresses into more specific lesions including diffuse vasculopathy and microthrombi, which is related to clinical neuropsychiatric symptoms (Cohen et al., 2017). Therefore, hypothetically, NPSLE is considered the consequence of these cumulative pathological damages to the nervous system when exceeding a certain threshold (Petri et al., 2008; Kozora et al., 2012; Cohen et al., 2017; Schwartz et al., 2019). On the basis of these findings and hypothesis, we speculated that the GM within the left temporal lobe including the L-STG may be one of the direct targets of lupus-related inflammatory attack, in which it exhibited a progressive pathological change.

In accordance with previous fMRI studies (Desmond et al., 2003; Hester and Garavan, 2004; Ren et al., 2012), we also found that (Figure 2 and Table 2), to compensate for the cerebral regional dysfunction, the areas in cerebellar posterior lobes were significantly activated in patients with non-NPSLE. Thus, cerebellar posterior lobes, an area which has a role in working memory, language processing and other executive tasks and is also included within the DMN (Schmahmann et al., 2019), may play a central role in the adaptability and plasticity of the brain to limit the functional impairment that has been caused by the disease. However, in patients with NPSLE as we found, while the GM volume within the cerebellar posterior lobes significantly reduced, its functional activities were attenuated as well, with increased FC of the ROIs within the posterior DMN and the regions around the L-STG. Increased cerebellar RS FC has been found in many neurological (Simioni et al., 2016) and neuropsychiatric (Feng et al., 2017) diseases. Bonacchi et al. (2020) identified that higher RS FC in the left cerebellar crus I was associated with worse memory performance. Taken together, these findings indicated a probable maladaptive rewiring and a trend of decompensation of the cerebellum to the disease damage in the state of NPSLE (Nystedt et al., 2019). In addition, other studies have demonstrated that compensatory functional signals decreased in SLE patients with disease duration of more than 10 years, indicating that the compensatory activations could be weakened by irreversible neural injuries (Mackay et al., 2011).

In line with the recently published literature (Bonacchi et al., 2020), the cerebral clusters in which we observed significant functional alterations during resting state in patients with NPSLE as compared with controls were the bilateral middle cingulate, the left superior temporal gyri, the bilateral cerebellar posterior lobes and the left medial superior frontal gyri within DMN, the right insula and the left anterior cingulate within salience network (SN), and the precentral and postcentral gyri within sensorimotor network (SMN). These neural networks are organized in balance. Previous research have verified the DMN and the SMN are anti-correlated, both regulated by the SN (Fox et al., 2005; Huang et al., 2015; Wang et al., 2019; Russo et al., 2020). The SN plays a critical role in attention and attributing saliency to external or internal originated events or stimuli and thus exerts control and balance on the DMN and other networks including the SMN (Shott et al., 2012; Martino et al., 2016). During resting state, the DMN is activated while the SMN is inhibited through the SN in healthy subjects. In patients with NPSLE, we observed attenuation of the DMN and the SN, and increased activation of the SMN during resting state. DMN abnormalities have been consistently verified in several neurological (Hohenfeld et al., 2018; Valsasina et al., 2019; Preziosa et al., 2020) and psychiatric (Chahine et al., 2017) disorders. As previous research reported (Barraclough et al., 2019), the DMN was indicated to have attenuated deactivation during performing cognitive tasks, and hypoconnectivities within it and between the cognitive networks during resting state (Nystedt et al., 2018) in NPSLE patients, which was interpreted as a compensatory mechanism resulting in preserved cognitive performance. Meanwhile, as previous fMRI studies reported, the RS FC within SN is severely impaired in major depression (Philip et al., 2018). However, Bonacchi et al. (2020) found increased RS FC in the left insular cortex with more severe depression, and decreased RS FC in the right anterior cingulate cortex with better memory performance within SN, suggesting an adaptive mechanism, probably contributing to a more efficient performance of the SN on cognitive tasks. Taken together, we speculated that the increased function of the nodes within SMN might be the consequence of the disinhibition of it, due to weakened control and regulation from the attenuated SN and DMN (Lin et al., 2011; Nystedt et al., 2018; Papadaki et al., 2018), which was also reported in previous studies (Nystedt et al., 2018), and may partially explain the inattention, hyperactivity and impulsivity in patients with NPSLE as reported in prior literature (Garcia et al., 2013; Gao et al., 2015).

By correlation analysis, we verified that the widespread GM atrophy in patients with NPSLE was negatively correlated with SDI scores. Meanwhile, the subcortical GM atrophy was negatively correlated with lesion volume (Table 3). These results suggested that the distribution and the degree of brain GM atrophy in NPSLE patients could be an indicator of disease burden (Mak et al., 2016; Liu et al., 2018). In addition, we found that the characteristic functional alterations in the cerebellar posterior lobes and the sensorimotor center as detailed above were associated with disease duration, SDI scores, lesion volume, hypertension, and aPLs, which was consistent with previous evidence, reflecting a reduction of the brain adaptability to maintain normal function along with severe pathologic burden and multiple cerebral vascular risk factors (Rocca et al., 2006; Mackay et al., 2011; Cohen et al., 2017; Papadaki et al., 2018). However, it is worth noting that the functional alterations in GM within these regions was negatively associated with cumulative corticosteroid use, which might suggest a therapeutic effect for corticosteroids and requires further study.

In addition, the SVM analysis (Figure 3 and Table 4) further confirmed that the combination of the ReHo of the bilateral cerebellar posterior lobes may be a potential imaging biomarker in early diagnosis of NPSLE, with both sensitivity and specificity above 0.8. Our results provided relatively satisfactory proof of the notion that the diagnostic process of NPSLE could be aided by objective MRI imaging parameters rather than merely physician assessment (Zirkzee et al., 2012; Fanouriakis et al., 2016).

There are some limitations in our study. First, the sample size is relatively small. However, sample sizes of this magnitude have been confirmed to have adequate signal sensitivity to obtain statistical significance as a pilot study. Certainly, further studies are needed to confirm the present observations on a larger sample, with longitudinal follow-up and assistance of neuropsychiatric assessments. Second, the patients recruited in our study had all received treatment with steroids and immunosuppressors. However, this limitation is inherent to this type of study. Third, the educational level of the subjects should be considered as a potential confounder in the future study. Forth, there were around 1/3 patients with active NPSLE, which might probably influence the results due to active inflammation. However, the median SLEDAI scores and the rate of patients with SLEDAI score ≥ 5 between the NPSLE and non-NPSLE groups had no significant difference, which had balanced the potential confounder. Fifth, the patients enrolled in the NPSLE group were heterogeneous, according to the different NP manifestations the patients had. However, in the present pilot study, we aimed to investigate the overall characteristics of the GM in patients with NPSLE, and we planned to refine the patients with NPSLE in the future study. Despite its exploratory nature, this quantitative MRI pilot study offers valuable insights into the brain reorganizational capacity in SLE patients, as well as indicates for the first time the functional parameter of the cerebellar posterior lobes may be a potential imaging biomarker to aid the early diagnosis of NPSLE. Further investigation of the underlying lupus-related vascular damage and BBB impairment in SLE patients using other advanced quantitative MRI techniques (e.g., high-resolution MR angiography, MR permeability imaging, susceptibility weighted imaging, and quantitative susceptibility mapping) is, therefore, an essential next step.

## Conclusion

In this study, characteristic deep nuclei atrophy and functional alteration pattern in GM within brain networks were identified in patients with NPSLE as compared with matched groups, which especially involved the cognitive and sensorimotor regions, and mainly associated with disease burden and aPLs. The different forms of the relationship between the structural and functional changes in patients with NPSLE and non-NPSLE reflected a compensatory mechanism of the brain to maintain normal function, which was attenuated along with pathologic burden and cerebral vascular risk factors. We also, for the first time, demonstrated *in vivo* that the GM within the left temporal lobe may be one of the direct targets of lupus-related inflammatory attack. Finally, we found that the function of the cerebellar posterior lobes might play an essential role in compensating for cortical functional disturbances and may contribute to identifying patients with suspected NPSLE in clinical practice. Larger longitudinal studies are required to further validate these data.

## Data Availability Statement

The original contributions presented in the study are included in the article/supplementary material, further inquiries can be directed to the corresponding author.

## Ethics Statement

The studies involving human participants were reviewed and approved by the Ethics Committee at the Peking Union Medical College Hospital. The patients/participants provided their written informed consent to participate in this study.

## Author Contributions

LS was responsible for the study design, patient recruit, data acquisition, and manuscript drafting. ZZ was responsible for the data analysis and manuscript drafting. YD, JH, XQ, and ML help the data acquisition and manuscript editing. YL and XZ were responsible for the study design, manuscript review, and final approval of this manuscript. All authors contributed to the article and approved the submitted version.

## Conflict of Interest

The authors declare that the research was conducted in the absence of any commercial or financial relationships that could be construed as a potential conflict of interest.

## Publisher’s Note

All claims expressed in this article are solely those of the authors and do not necessarily represent those of their affiliated organizations, or those of the publisher, the editors and the reviewers. Any product that may be evaluated in this article, or claim that may be made by its manufacturer, is not guaranteed or endorsed by the publisher.

## Footnotes

1
http://rfmri.org/DPARSF

2
http://www.nitrc.org/projects/conn

3
https://www.fil.ion.ucl.ac.uk/spm/

4
http://www.csie.ntu.edu.tw/~cjlin/libsvm/
